# Survival outcomes of low prostate-specific antigen levels and T stages in patients with high-grade prostate cancer: a population-matched study

**DOI:** 10.7150/jca.40428

**Published:** 2020-09-17

**Authors:** Yongming Kang, Pan Song, Kun Fang, Bo Yang, Luchen Yang, Jing Zhou, Linchuan Wang, Qiang Dong

**Affiliations:** 1 Department of Urology, Institute of Urology, West China Hospital, Sichuan University, Chengdu, 610041, Sichuan Province, China.; 2 Department of Urology, Suining Central Hospital, Suining, 629000, Sichuan Province, China.

**Keywords:** Prostate cancer, Gleason score, Prostate-specific antigen, T stage, Prognosis

## Abstract

**Objective:** To evaluate the prostate cancer-specific survival (PCSS) of low T stages or low prostate-specific antigens (PSA) levels in men with high-grade prostate cancer.

**Materials and Methods:** Patients with non-metastatic prostate cancer (T1-4N0M0) and Gleason score 8-10 in the Surveillance, Epidemiology, and End Results database from 2004-2010 were identified. These men were stratified by T stages (T1, T2, T3a, T3b-4) and PSA levels (<4.0 ng/ml, 4.0-10.0 ng/ml, 10.1-20.0 ng/ml, >20.0 ng/ml). Propensity-score matching (PSM) was conducted to balance the covariates. Kaplan-Meier analysis and multivariable Cox regressions were performed to analyze the PCSS in different T stage or PSA levels groups.

**Results:** A total of 33231 patients aging 69(62~76) years were identified. The overall cohort results showed that the PCSS of T1 group was significantly worse than that of T2 and T3a groups [T2 HR: 0.62(0.57~0.67); T3 HR: 0.70(0.63~0.77)]. There were no significant difference between T2 and T3a groups [T2 HR: 0.98 (0.91~1.05)]. The PSA <4.0 ng/ml group had significantly worse PCSS than PSA 4.0-10.0 ng/ml [PSA 4.0-10.0 ng/ml HR: 0.77(0.68~0.88)]. PSM methods were implemented in the comparison of T1 vs T2, T1 vs T3a, T2 vs T3a. and PSA< 4.0 ng/ml vs PSA 4.0-10.0 ng/ml, The results in these matched cohorts showed that T1 group was associated with significantly worse PCSS than T2 group [T1 HR: 1.31(1.20~1.44)] and T3a group [T1 HR: 1.33(1.16~1.52)]. There were no significant differences between T2 and T3a groups [T3a HR: 1.14(0.99~1.32)]. The PCSS of patients with PSA< 4.0 ng/ml was significantly worse that these with PSA 4.0-10.0 ng/ml in the matched cohort [PSA< 4.0 ng/ml HR: 1.3(1.08~1.56)].

**Conclusions:** For patients with high-grade PCa, the PCSS of patients seems to be worse in the T1 stage than those in T2 and T3a stages. Patients with PSA <4.0 ng/ml appears to have poorer prognosis than those with PSA 4.0-10.0 ng/ml.

## Introduction

Prostate cancer (PCa) is regarded as the most common malignant tumor of the male urogenital system and the second cause of cancer-related death in men, seriously threatening the life and health of patients in the world 1, 2. It is estimated that, in 2020, there will be approximately 191,930 newly diagnosed men with PCa and 33,330 cases will be dead for PCa 3. Due to the great harm of PCa, it is necessary to predict the prognosis of PCa in advance and distinguish the one with poor prognosis. Nowadays, Gleason score (GS), prostate-specific antigen (PSA) and clinical Tumor Node Metastasis (TNM) stages are considered as the most important factors that have significant impacts on the prognosis of PCa and the choices of treatments 4. GS system reflects the histological classification of tumors. High-grade disease (GS 8-10) is an important indicator of the prognosis of patients with non-metastatic PCa 5, but might have fewer effects on the indication of metastatic lesions 6, 7. The levels of pretreatment serum PSA reveal the burden of tumor cells, and have close relationships with the prognosis of patients. The TNM stages measure the size of the tumor and the extent of invasion, as well as metastasis. For non-metastatic PCa, all these factors are closely related to the risk classification and prognosis of PCa.

Though many novel predictors for the prognosis of PCa have been reported 8-10, the factors of GS, PSA levels and TNM stages remain the most commonly used indicators for the prognosis of PCa. Generally, patients with higher GS, PSA levels or TNM stages are associated with worse prostate cancer-specific survival (PCSS) 11. However, there are some exceptions in the high-grade PCa according to the previously published articles. It was reported that patients with high-grade PCa and low PSA levels seem to have reduced survival outcomes 12-14. As few studies have explored the prognosis of men with different T stages in high-grade diseases, it is unclear whether there are exceptions in the comparison of T stages in men with high-grade diseases. The aim of this study was to evaluate the prognostic differences among T stages (T1, T2, T3a, T3b-4) and PSA levels (PSA <4.0 ng/ml, 4.0-10.0 ng/ml, 10.1-20.0 ng/ml, 20.1-40.0 ng/ml, >40 ng/ml) in patients with non-metastatic high grade (T1-4N0M0 GS 8-10) PCa.

## Materials and methods

### Data source

The data of this study were derived from the Surveillance, Epidemiology and End Results (SEER) database with the software SEER* STAT. Patients with high-grade PCa diagnosed from January 1, 2004 to December 31, 2010 were retrospectively identified.

### Selection criteria

Inclusion criteria: (1) patients were diagnosed with non-metastatic PCa (cT1-4N0M0). (2) High-grade (GS 8-10) was detected by needle core biopsy, transurethral resection of the prostate, or prostatectomy. (3) The PSA levels, clinical T stage were clearly known.

Exclusion criteria: (1) multiple tumor; (2) important information like age, follow-up time was unclear or incomplete. (3) The cancer-specific survival states at the end of follow-ups were unclear.

### Variables and main outcomes

The following baseline characteristics were collected including age (<65, 65-75, >75), race (white, black, other race including American Indian and Asian/Pacific Islander), marital status (married, unmarried, divorced or separated), T stage (T1, T2, T3a, T3b-4), PSA levels (<4.0 ng/ml, 4.0-10.0 ng/ml, 10.1-20.0 ng/ml. >20.0 ng/ml), Gleason score (8,9-10), therapy (local treatments including radical prostatectomy and external beam radiotherapy, no local treatments), survival months, and prostate cancer-specific survival status (alive, dead for PCa, dead for other reasons). PCSS was regarded as the main outcome.

### Statistical analyses

Baseline characteristics in different PSA level groups (<4.0 ng/ml, 4.0-10.0 ng/ml, 10.1-20.0 ng/ml, and >20.0 ng/mL) and T stage groups (T1, T2, T3a, T3b-4) were described. Kaplan-Meier analysis was introduced to assess the PCSS in different PSA levels and T stages groups and the survival curves were constructed. Univariate and multivariate Cox analysis were used to calculate hazard ratios (HR) and its 95% confidence interval (95% CI). For groups with similar PCSS results in the overall cohort, propensity-score matching (PSM) based on the nearest-neighbor matching principle was adopted to balance the covariates and generate the matched cohorts. The PCSS was reevaluated in the matched groups to verify the results in overall cohort. All statistical analyses were conducted in the software of SPSS 25.0 and Graph prism 7.0.

## Results

### Patients' characteristics

33,231 patients with a median age of 69 (62-76) years were included. The median follow-up time was 82 (62~109) months. The baseline characteristics of the included patients in PSA level groups and T stage groups were presented in **Table 1**. The PSM was conducted in the comparison of T1 vs T2, T1 vs T3a, T2 vs T3a and PSA <4.0 ng/ml vs PSA 4.0-10.0 ng/ml group. The basic characteristics of matched groups were shown in **Table 2.**

### Survival outcomes

#### PCSS of patients in different T stage groups

**Figure 1:**
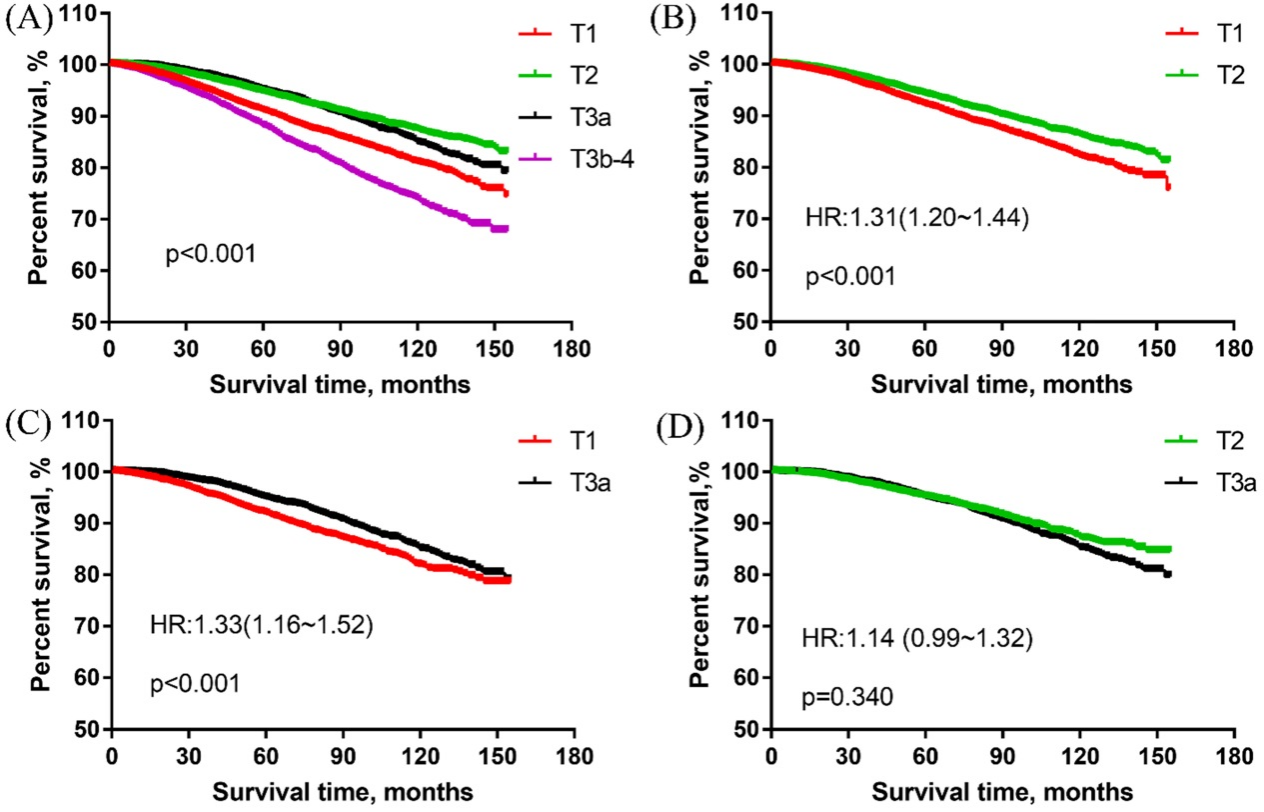
The prostate cancer-specific survival of men with high-grade and different T stage prostate cancer. (A) The prostate cancer-specific survival in overall cohort. (B) The prostate cancer-specific survival in the matched cohort of T1 vs T2 group. (C) The prostate cancer-specific survival in the matched cohort of T1 vs T3a group. (D) The prostate cancer-specific survival in the matched cohort of T2 vs T3a group.

In overall cohort, the survival curves revealed that the T1 group had significantly worse PCSS than the T2 group and T3a group for men with high-grade PCa. With T1 as the reference, the HRs and 95%CIs of T2, T3a, T3b-4 were 0.62(0.57~0.67), 0.70(0.63~0.77) and 1.41(1.30~1.53), respectively. T2 group was associated with the best PCSS but no obvious difference existed between T2 and T3a group [HR: 0.98 (0.91~1.05)]. The T3b-4 group had the worst survival results among all T stage groups. These results were presented in **Figure 1A**.

PSM was conducted in the comparison of T1 vs T2 group, T1 vs T3a group. The matched groups were analyzed to verify the results of the overall cohort. There were 16,794 patients matched in the comparison of T1 vs T2 group. The PCSS results showed that T1 group was significantly worse than T2 group (**Figure 1B**). With T2 as the reference, the HR and 95%CI of T1 was 1.31 (1.20~1.44).

A total of 7348 patients were left in the matched group of T1 vs T3a, the PCSS results revealed that T1 group was obviously worse than T3a group for men with high-grade PCa. With T3a as the reference, the HR and 95%CI of T1 was 1.33 (1.16~1.52) (**Figure 1C**).

In the matched group of T2 vs T3a group, there were 7668 patients. As shown in **Figure 1D**, there were no significant differences between T2 and T3a groups in the matched cohort. With T2 as the reference, the HR and 95%CI of T3a was 1.14 (0.99~1.32).

#### PCSS of patients in different PSA level groups

**Figure 2:**
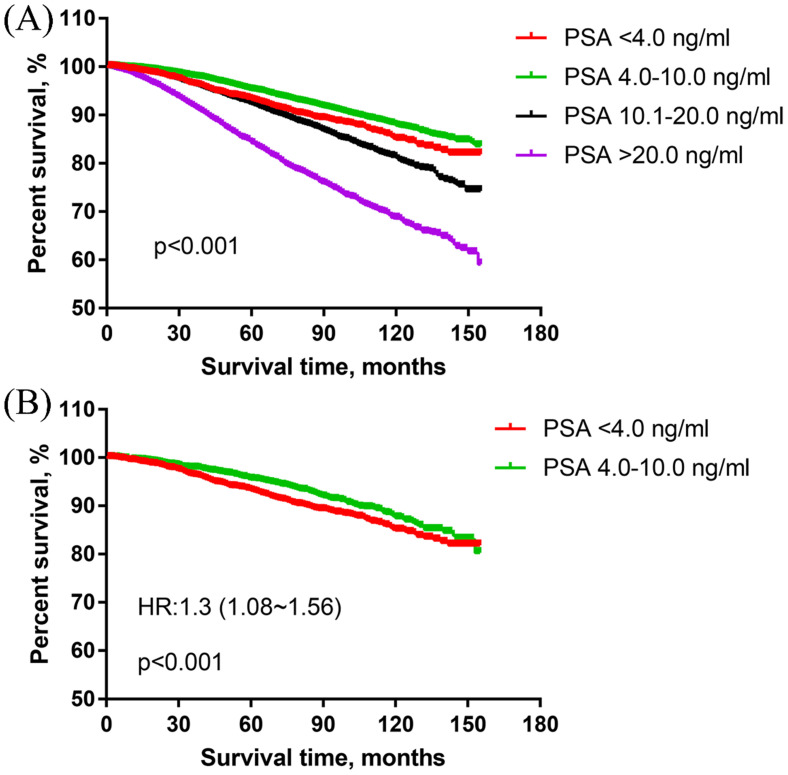
The prostate cancer-specific survival of men with high-grade and different PSA levels PCa. (A) The prostate cancer-specific survival in overall cohort. (B) The prostate cancer-specific survival in the matched cohort of PSA<4.0 ng/ml vs PSA4.0-10.0 ng/ml group.

In the overall cohort, the survival curve showed that the PSA 4.0-10.0 ng/ml group had obviously better PCSS than PSA <4.0 ng/ml group. PSA <4.0 ng/ml group was associated with significantly better PCSS than PSA 10.1-20.0 ng/ml and PSA >20.0 ng/ml. With PSA <4.0 ng/ml as the reference, the HRs and 95% CIs of PSA 4.0-10.0 ng/ml, 10.1-20.0 ng/ml, >20.0 ng/ml were 0.77 (0.68~0.88), 1.27 (1.11~1.46) and 2.44 (2.14~2.79), respectively. The results were presented in **Figure 2A**.

In the matched cohort, there were 2323 patients in each of PSA <4.0 ng/ml and PSA 4.0-10.0 ng/ml group. The PCSS results showed that PSA <4.0 ng/ml group was associated with significantly worse PCSS than PSA 4.0-10.0 ng/ml group in patients with high-grade PCa [HR: 1.30 (1.08~1.56)] (**Figure 2B**).

#### Multivariate COX analysis for PCSS

Multivariate COX analysis results of PCSS were presented in **Table 3**. Factors related to the risk of cancer-specific death included age, race, marital status, T stage, Gleason score, PSA, and treatments. With T1 as the reference, The HRs of T2, T3a and T4 for the cancer-specific mortality of men with high-grade PCa were 0.78 (0.72~0.84), 0.86 (0.78~0.96) and 1.39 (1.27~1.52), respectively. With PSA < 4.0 ng/ml as the reference, the HR of PSA 4.0-10.0 ng/ml, PSA 10.1-20.0 ng/ml, and PSA >20.0 ng/ml for cancer-specific mortality of men with high-grade PCa were 0.79 (0.69~0.9), 1.09 (0.95~1.25) and 1.84 (1.61~2.1), respectively.

## Discussion

The factors of GS, PSA or T stage are the widely accepted risk-grading standards in non-metastatic prostate cancer and have great impacts on the treatment decisions 15. High-grade PCa has a great impact on the prognosis of patients and has always been one of the focuses in PCa researches 16, 17. In general, PCa with higher PSA levels or T stages is more aggressive and associated with poorer prognosis for the patients. However, there seem to be some exceptions for high-grade GS PCa.

In our study, 33231 patients with high-grade PCa were grouped according to their PSA levels (<4.0 ng/ml, 4.0-10.0 ng/ml, 10.1-20.0 ng/ml, >20.0 ng/ml) and T stages (T1, T2, T3a and T3b-T4). The results in T stage groups showed that men with T1 stage had significantly worse PCSS than those with T2 stage or even T3a stage. The survival differences between T2 and T3a group were not obvious. The PCSS results in the matched groups also showed that T1 group was worse than T2 and T3a groups. Our results revealed that patients with T1 stage and high-grade PCa seem to have poorer cancer-specific survival than those with T2 stage or T3a stage. As far as we can know, these results haven't been reported in previous studies. High-grade PCa with low T stages might be associated with a feature of increased aggressive behavior. However, these results might be disturbed by the inaccurate clinical T staging in clinical practices. As T stage does not have enough prognostic values for localized PCa, the T stage of many patients might be assessed inaccurately. Therefore, the reliability of these results should be evaluated carefully in further studies.

In the comparison of PSA level groups, the results in the overall cohort showed that PSA 4.0-10.0 ng/ml group had the best PCSS among all level groups. PSA <4.0 ng/ml group was significantly worse than PSA 4.0-10.0 ng/ml group. The verification of matched cohorts also showed similar results. Patients in PSA 4.0-10.0 ng/ml group were associated with obviously better PCSS than those in PSA <4.0 ng/ml group in the matched cohort. These results have been revealed by several previous studies In Falchook's study 18, they found that patients with high-grade PCa and PSA<4.0 ng/ml were associated with poorer survival outcomes than those with PSA 4-9.9 ng/ml. Mahal et al. 19 reported that high-grade PCa with low PSA levels was especially aggressive. Men with high-grade PCa and PSA 2.6-4.0 ng/ml were associated with poorer prognoses than those with PSA 4.0-10.0 ng/ml, but obviously better survival outcomes than those with PSA <2.5 ng/ml. Several studies 14, 19-22 have proposed a hypothesis that high-grade PCa with low PSA level has the characteristics of dedifferentiation, clinically aggressiveness, and hormone resistance, but additional evidence are needed for further confirmation.

Our results were conducted with a large number of patients and were verified by the PSM groups. However, there were still some limitations in our study. Firstly, our study was a retrospective study, many confounding factors existed even in the matched groups. Therefore, it's inevitable to get influenced by some potential biases. Secondly, our data was only derived from the SEER database without the validation of our own data. It led to our results lacking sufficient persuasion. Thirdly, limited by the data characteristics in the open database, we only included the available factors in the database. Some important covariates might be missed in our analysis. It might have an impact on our results. Therefore, high-quality studies are needed in the future to verify the results.

## Conclusion

High-grade PCa with low T stages or low PSA levels seems to be particularly aggressive and patients with these indicators are associated with decreased PCSS.

## Figures and Tables

**Table 1:** Baseline characteristics of patients in different T stage groups and PSA groups

Characteristic	Total	T1	T2	T3a	T3b-T4	PSA <4.0 ng/ml	PSA 4.0-10.0 ng/ml	PSA 10.1-20.0 ng/ml	PSA >20.0 ng/ml
N	33231	15131	9759	4187	4154	2336	16309	7640	6946
**Age (years)**									
Median (IQR)	69 (62~76)	73 (66~78)	68 (62~75)	64 (59~69)	65 (59~71)	68 (61~74)	68 (62~74)	71 (64~77)	71 (63~78)
**Age, n (%)**									
<65	11962 (36)	3576 (23.6)	3839 (39.3)	2383 (56.9)	2164 (52.1)	955 (40.9)	6343 (38.9)	2409 (31.5)	2255 (32.5)
65-75	12696 (38.2)	6041 (39.9)	3695 (37.9)	1484 (35.4)	1476 (35.5)	883 (37.8)	6709 (41.1)	2796 (36.6)	2308 (33.2)
>75	8573 (25.8)	5514 (36.4)	2225 (22.8)	320 (7.6)	514 (12.4)	498 (21.3)	3257 (20)	2435 (31.9)	2383 (34.3)
**Race, n (%)**						955 (40.9)	6343 (38.9)	2409 (31.5)	2255 (32.5)
White	25441 (76.6)	10980 (72.6)	7613 (78)	3491 (83.4)	3357 (80.8)				
Black	5220 (15.7)	2916 (19.3)	1413 (14.5)	394 (9.4)	497 (12)	1980 (84.8)	12866 (78.9)	5748 (75.2)	4847 (69.8)
Others	2274 (6.8)	1060 (7)	661 (6.8)	281 (6.7)	272 (6.5)	246 (10.5)	2260 (13.9)	1194 (15.6)	1520 (21.9)
Unclear	296 (0.9)	175 (1.2)	72 (0.7)	21 (0.5)	28 (0.7)	94 (4)	1028 (6.3)	627 (8.2)	525 (7.6)
**Marriage, n (%)**									
Married	22885 (68.9)	9628 (63.6)	6966 (71.4)	3237 (77.3)	3054 (73.5)	1745 (74.7)	11799 (72.3)	5177 (67.8)	4164 (59.9)
Unmarried	3030 (9.1)	1438 (9.5)	867 (8.9)	343 (8.2)	382 (9.2)	178 (7.6)	1324 (8.1)	720 (9.4)	808 (11.6)
Separated	4644 (14)	2347 (15.5)	1356 (13.9)	450 (10.7)	491 (11.8)	254 (10.9)	2018 (12.4)	1139 (14.9)	1233 (17.8)
Unclear	2672 (8)	1718 (11.4)	570 (5.8)	157 (3.7)	227 (5.5)	159 (6.8)	1168 (7.2)	604 (7.9)	741 (10.7)
**T stage, n (%)**									
T1	15131 (45.5)	15131 (100)	-	-	-	681 (29.2)	7019 (43)	3796 (49.7)	3635 (52.3)
T2	9759 (29.4)	-	9759 (100)	-	-	963 (41.2)	5206 (31.9)	1954 (25.6)	1636 (23.6)
T3a	4187 (12.6)	-	-	4187 (100)	-	370 (15.8)	2319 (14.2)	869 (11.4)	629 (9.1)
T3b-4	4154 (12.5)	-	-	-	4154 (100)	322 (13.8)	1765 (10.8)	1021 (13.4)	1046 (15.1)
**PSA, n (%)**									
<4.0 ng/ml	2336 (7)	681 (4.5)	963 (9.9)	370 (8.8)	322 (7.8)	2336 (100)	-	-	-
4.0-10.0 ng/ml	16309 (49.1)	7019 (46.4)	5206 (53.3)	2319 (55.4)	1765 (42.5)	-	16309 (100)	-	-
10.1-20.0 ng/ml	7640 (23)	3796 (25.1)	1954 (20)	869 (20.8)	1021 (24.6)	-	-	7640 (100)	-
>20.0 ng/ml	6946 (20.9)	3635 (24)	1636 (16.8)	629 (15)	1046 (25.2)	-	-	-	6946 (100)
**Gleason score, n (%)**									
8	19447 (58.5)	9774 (64.6)	6001 (61.5)	2052 (49)	1620 (39)	1344 (57.5)	10207 (62.6)	4357 (57)	3539 (51)
9-10	13784 (41.5)	5357 (35.4)	3758 (38.5)	2135 (51)	2534 (61)	992 (42.5)	6102 (37.4)	3283 (43)	3407 (49)
**Therapy, n (%)**									
Local treatments	24972 (75.1)	10635 (70.3)	8282 (84.9)	3286 (78.5)	2769 (66.7)	1870 (80.1)	13319 (81.7)	5599 (73.3)	4184 (60.2)
No local treatments	8259 (24.9)	4496 (29.7)	1477 (15.1)	901 (21.5)	1385 (33.3)	466 (19.9)	2990 (18.3)	2041 (26.7)	2762 (39.8)
**Survival time (months)**									
Median (IQR)	82 (62~109)	77 (56~104)	86(64~113)	92 (70~117)	82.5 (62~109)	86.5 (65~113)	87 (66.5~113)	79 (60~107)	72 (43~100)

IQR, interquartile range; GS: Gleason score; PSA, prostate-specific antigen.

**Table 2:** Baseline characteristics of patients in propensity-score matched groups

Characteristic	T1	T2	*p*	T1	T3a	*p*	T2	T3a	*p*	PSA <4.0ng/ml	PSA 4.0-10.0 ng/ml	*p*
N	8397	8397		3674	3674		3834	3834		2323	2323	
**Age (years)**												
Median (IQR)	70 (64~76)	70 (62~74)		65 (63~75)	65 (61~70)		65 (61~72)	65 (60~70)		68 (61~74)	72 (65~75)	
**Age, n (%)**												
<65	2735 (32.6)	2733 (32.5)	0.999	1876 (51.1)	1885 (51.3)	0.828	2084 (54.4)	2088 (54.5)	0.964	951 (40.9)	951 (40.9)	1.000
65-75	3462 (41.2)	3465 (41.3)		1464 (39.8)	1470 (40)		1429 (37.3)	1427 (37.2)		879 (37.8)	879 (37.8)	
>75	2200 (26.2)	2199 (26.2)		334 (9.1)	319 (8.7)		321 (8.4)	319 (8.3)		493 (21.2)	493 (21.2)	
**Race, n (%)**												
White	6456 (76.9)	6456 (76.9)	0.990	3034 (82.6)	3046 (82.9)	0.836	3224 (84.1)	3228 (84.2)	0.990	1978 (85.1)	1968 (84.7)	0.930
Black	1372 (16.3)	1369 (16.3)		403 (11)	391 (10.6)		366 (9.5)	366 (9.5)		238 (10.2)	239 (10.3)	
Others	529 (6.3)	529 (6.3)		226 (6.2)	222 (6)		236 (6.2)	231 (6)		92 (4)	101 (4.3)	
Unknown	40 (0.5)	43 (0.5)		11 (0.3)	15 (0.4)		8 (0.2)	9 (0.2)		15 (0.6)	15 (0.6)	
**Marriage, n (%)**												
Married	5857 (69.8)	5857 (69.8)	0.999	2786 (75.8)	2789 (75.9)	0.663	2952 (77)	2960 (77.2)	0.952	1741 (74.9)	1740 (74.9)	0.927
Unmarried	762 (9.1)	759 (9)		306 (8.3)	305 (8.3)		316 (8.2)	308 (8)		174 (7.5)	174 (7.5)	
Separated	1240 (14.8)	1238 (14.7)		447 (12.2)	427 (11.6)		427 (11.1)	420 (11)		251 (10.8)	261 (11.2)	
unclear	538 (6.4)	543 (6.5)		135 (3.7)	153 (4.2)		139 (3.6)	146 (3.8)		157 (6.8)	148 (6.4)	
T stage, n (%)												
T1	8397 (100)	-	-	3674 (100)	-	-	-	-	-	679 (29.2)	669 (28.8)	0.989
T2	-	8397 (100)		-	-		3834 (100)	-		958 (41.2)	968 (41.7)	
T3a	-	-		-	3674 (100)		-	3834 (100)		367 (15.8)	367 (15.8)	
T3b-4	-	-		-	-		-	-		319 (13.7)	319 (13.7)	
PSA, n (%)												
<4.0 ng/ml	504 (6)	506 (6)	1.000	253 (6.9)	249 (6.8)	0.990	324 (8.5)	325 (8.5)	1.00	2323 (100)	-	-
4.0-10.0 ng/ml	4449 (53)	4452 (53)		2047 (55.7)	2038 (55.5)		2146 (56)	2148 (56)		-	2323 (100)	
10.1-20.0 ng/ml	1827 (21.8)	1827 (21.8)		761 (20.7)	767 (20.9)		775 (20.2)	772 (20.1)		-	-	
>20.0 ng/ml	1617 (19.3)	1612 (19.2)		613 (16.7)	620 (16.9)		589 (15.4)	589 (15.4)		-	-	
Gleason score, n (%)												
8	5224 (62.2)	5221 (62.2)	0.962	1999 (54.4)	2003 (54.5)	0.925	1956 (51)	1954 (51)	0.964	1341 (57.7)	1333 (57.4)	0.812
9-10	3173 (37.8)	3176 (37.8)		1675 (45.6)	1671 (45.5)		1878 (49)	1880 (49)		982 (42.3)	990 (42.6)	
Therapy, n (%)												
Local treatments	6954 (82.8)	6956 (82.8)	0.967	2917 (79.4)	2934 (79.9)	0.622	3236 (84.4)	3241 (84.5)	0.857	1867 (80.4)	1875 (80.7)	0.767
No local treatments	1443 (17.2)	1441 (17.2)		757 (20.6)	740 (20.1)		598 (15.6)	593 (15.5)		456 (19.6)	448 (19.3)	
Survival time (months)												
Median (IQR)	85 (63~111)	89 (65~112)		80 (61~109)	91 (70~116)		87 (66~114)	91 (70~116)		86 (65~113)	85 (64~113)	

IQR, interquartile range; GS: Gleason score; PSA, prostate-specific antigen.

**Table 3:** Multivariate COX analysis for prostate cancer-specific survival in overall cohort

Risk factors	PCSS		
	HR	95% CI	*P*
**Age**			
<65	1		Ref.
65-75	1.22	(1.13~1.32)	< 0.001
>75	1.81	(1.67~1.97)	< 0.001
**Race**			
White	1		Ref.
Black	1.2	(1.1~1.3)	< 0.001
Others	0.78	(0.69~0.89)	< 0.001
**Marital status**			
Married	1		Ref.
Unmarried	1.3	(1.18~1.45)	< 0.001
Separated	1.31	(1.2~1.42)	< 0.001
**T stage**			
T1	1		Ref.
T2	0.78	(0.72~0.84)	< 0.001
T3a	0.86	(0.78~0.96)	0.006
T3b-4	1.39	(1.27~1.52)	< 0.001
**PSA**			
< 4.0 ng/ml	1		Ref.
4.0-10.0 ng/ml	0.79	(0.69~0.9)	< 0.001
10.1-20.0 ng/ml	1.09	(0.95~1.25)	0.221
> 20.0 ng/ml	1.84	(1.61~2.1)	< 0.001
**Gleason score**			
8	1		Ref.
9-10	2.09	(1.96~2.22)	< 0.001
**Treatment**			
Local treatments	1		Ref.
No local treatments	1.76	(1.65~1.88)	< 0.001

HR, hazard ratio; Ref, reference; PSA, prostate-specific antigen; 95% CI: 95% confidence interval.
